# Platelet-rich plasma (PRP) therapy: An approach in reproductive medicine based on successful animal models

**DOI:** 10.21451/1984-3143-AR2018-093

**Published:** 2020-05-22

**Authors:** Natalia Juliana Nardelli Gonçalves, Nilo Frantz, Ricardo Manuel de Oliveira

**Affiliations:** RDO Medical Diagnosis, São Paulo, Brazil.

**Keywords:** Platelet-rich plasma, reproduction, uterine environment, infertility

## Abstract

Platelet-rich plasma (PRP) has been fully studied for different clinical applications in veterinary medicine for many years with promising results. As a result, therapeutic studies to elucidate pathways for PRP use in human reproduction have been performed. PRP applications in human reproductive medicine are recent, but the role of platelet growth factors in improving the endometrial environment is well known. Indications for PRP therapy show its positive effects in promoting endometrial and follicular growth and gestation in assisted reproduction cycles, as has been proven in animals. We summarized the putative role of PRP on endometrial receptivity with a brief history of promising results in research and clinical therapies.

## Introduction

Platelets play an important role in hemostasis by preventing blood loss at sites of vascular injury. The physiologic process begins with the adherence and aggregation of platelets that then forms a procoagulant surface, leading to thrombin generation and fibrin formation. Platelets also release substances that promote tissue repair and influence the reactivity of vascular and other blood cells in angiogenesis and inflammation. They contain important growth factors, such as PDGF (platelet-derived growth factor), TGF-β (transformer growth factor-β), and VEGF (vascular endothelial growth factor), as well as the cytokines PF4 (platelet factor 4) and CD40L (Sills *et al*., 2018; Anitua *et al*., 2004). These growth factors and cytokines are critical in the activation of fibroblasts and the recruitment of leukocytes to the injury site, inducing and regulating the proliferation and migration of other cell types involved in tissue repair. In addition, platelet-derived factors are essential for endometrial progenitor cell activity (Gargett *et al*., 2008), and PDGF isoforms significantly promote endometrial stromal cell proliferation, migration, and contractility (Matsumoto *et al*., 2005; Aghajanova *et al*., 2018).

Platelet-rich plasma (PRP) has been recently applied in reproductive scenarios and is based on the knowledge that platelet growth factors can improve the endometrial environment, which is full of growth-factor receptors, adhesion molecules, cytokines, lipids, and other factors that enhance endometrial and embryonic development. Despite the progress in the field of assisted reproductive technology, multiple embryos fail to implant.

PRP contains growth factors and other cytokines that have positive effects on local tissue repair and endometrial receptivity (Farimani *et al*., 2017). A significant percentage of in vitro fertilization failure is due to poor endometrial receptivity; implantation requires good embryo quality to provide a good coordination between mother and fetus. The human endometrium undergoes significant changes during implantation, and immune cells and their secreted substances in the luteal phase, such as granulocyte colony-stimulating factor (G-CSF), play an important role in this process (Farimani *et al*., 2017; Aghajanova *et al*., 2018).

In this context, the influence of platelet growth factors on the relationship between the uterine environment and embryo implantation has been widely studied, and the results are encouraging. We summarize the most relevant clinical findings in both animal models and clinical trials that led to the recognition of PRP as an important ally in reproductive medicine. 

## Platelet-rich plasma (PRP): a source of growth factors

PRP is easy to obtain, low cost, and rich in growth factors. Since PRP is an autologous preparation, and therefore non-toxic and non-allergenic, it can be used in various medical conditions as an adjuvant therapy to conventional treatment, with generally satisfactory results (Molina *et al*., 2018).

The use of autologous PRP in clinical research has grown exponentially over recent years due to the increasing understanding of the role PRP’s growth factors play in tissue regeneration (Scully *et al*., 2018). The history of PRP began with the first publication in Nature magazine (Kingsley, 1954), followed around ten years later by the first article to describe applying PRP in a therapeutic approach (Levin *et al*., 1964). As a result, PRP became an attractive methodology in several therapeutic areas.

**Figure 1:**
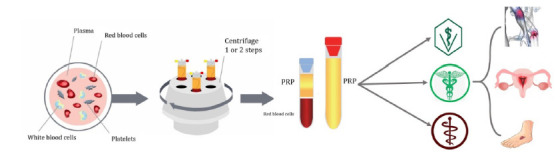
Illustration of platelet-rich plasma (PRP) protocol. Obtaining PRP involves the drawing of a small volume of blood from the patient, a centrifugation step to fractionate the blood, and the separation of platelet-rich fractions. Various formulations, including liquid clot and membrane, can be obtained for use in many applications, including veterinary medicine, human medicine, and odontology. In humans, PRP can be used especially in orthopedic issues, reproduction, and dermatological ulcerations.

New protocols, with the principal goal to improve the process and purity of PRP, are frequently published. Gutierrez *et al*. (2017) reported a two-step centrifugation protocol for concentrating cells and growth factors in bovine PRP. Figure 1 exemplifies the methodological basis for obtaining PRP and its many applications. Each preparation method is intended to create a product with a particular bioaction and, consequently, with a specific clinical application. Thus, PRP is not considered to be a single, final blood derivate containing plasma and high concentrations of platelets (Bos-Mikich *et al*., 2018). 

There are four classifications of platelet concentrates that depend on their leucocyte and fibrin content: pure platelet-rich plasma (P-PRP), leucocyte- and platelet-rich plasma (LPRP), pure platelet-rich fibrin (P-PRF), and leucocyte- and platelet-rich fibrin (L-PRF) (Ehrenfest *et al*., 2009; Gutierrez *et al*., 2017).

Bos-Mikich *et al*. (2018) described PRP as the volume of plasma that has a platelet count above the baseline. PRP was later defined as a group of soluble and diffusible polypeptide substances able to regulate the growth, differentiation, and phenotype of numerous cell types (Bos-Mikich *et al*., 2018). Platelets are small and anucleated cell fragments (2 to 3 μm in diameter) released from megakaryocytes found in the bone marrow (Pietrzak *et al*., 2005). 

We summarized the effects of PRP growth factors in Table 1, and the main ones are as follows: (1) PRP growth factors are capable of acting in a paracrine way on different cell types, such as myocytes, mesenchymal stem cells, chondrocytes, osteoblasts, and fibroblasts (Graziani *et al*., 2005; Anitua *et al*., 2009; Drengk *et al*., 2009; Cho *et al*., 2011; Van *et al*., 2011; Mazzocca *et al*., 2012); (2) PRP growth factors increase the infiltration of neutrophils and macrophages that promote angiogenesis, fibroplasia, and matrix deposition, which induce the consequent tissue regeneration; (3) and PRP growth factors have anti-inflammatory properties (Anitua *et al*., 2004; Bendinelli *et al*., 2010; Lange-Consiglio *et al*., 2015). 

**Table 1:** Growth factors secreted by platelets and their main functions.

TGF-β- **transforming growth factor beta**	PGDFa-b - platelet-derived growth factor
Undifferentiated mesenchymal cells prolifieration Regulates endothelial, fibroblastic, and osteoblastic mitogenesis Regulation of synthesis and secretion of collagen Regulates the mitogenic effect of other growth factors Chemotaxis and endothelial angiogenesis Inhibits proliferation of lymphocytes and macrophages	Mitogenic effect for mesenchymal cells and osteoblasts Mitogenesis/chemotaxis in fibroblasts and smooth muscle cells Regulates collagenase secretion and collagen synthesis Chemotaxis of neutrophils and macrophages
EGF- Epidermal growth factor	VEGF- Vascular endothelial growth factor
Angiogenesis and endothelial chemotaxis Regulates collagenase secretion Epithelial and mesenchymal mitogenesis	Increases angiogenesis and vascular permeability Stimulates mitogenesis to endothelial cells
bFGF- Basic fibroblast growth factor	CTGF - Connective tissue growth factor
Growth and differentiation of chondrocytes and osteoblasts Mitogenic factor for mesenchymal cells, chondrocytes, and osteoblasts	Angiogenesis and regeneration of cartilage Fibrosis and platelet aggregation

## PRP and successful animal models

PRP has been studied for several clinical applications in veterinary medicine with promising results, and this has encouraged therapeutic studies to elucidate the pathways for its use in human reproduction. Examples include using PRP in horses for orthopedic therapy (musculoskeletal lesions) and tenodesmic lesions (Scala *et al*., 2014; Brossi *et al*., 2015), and using PRP in dogs for orthopedic therapy of ligament rupture by intra-articular injections (Silva *et al*., 2013; Vilar *et al*., 2013). Groups of veterinary researchers have studied the biology of PRP more extensively (mostly cellular and molecular studies) in horses (Rinnovati *et al*., 2016; Giraldo *et al*., 2015; Hessel *et al*., 2015; Hauschild *et al*., 2017) than in dogs (Silva *et al*., 2012; Carr *et al*., 2016; Frye *et al*., 2016). 

Scully *et al*. (2018) reviewed the PRP applications in animal models for regenerative tissue, and they reported some important observations in rats (Hammond *et al*., 2009, Borrione *et al*., 2014; Martins *et al*., 2016; Li *et al*., 2016; Pinheiro *et al*., 2016; Borrione *et al*., 2017) using the application of PRP on a flexor sublimis lesion and tibialis anterior under muscle strain. The results showed a significant improvement in leukocyte infiltration, inflammatory response, the increase of pro-inflammatory cytokines, and myogenesis. Other studies of mouse and rat models using PRP reported an increase in cell proliferation, pro-inflammatory cytokines, and muscle regeneration (Notodihardjo *et al*., 2015; Denapoli *et al*., 2016). 

Larson *et al*. (1992) successfully developed bovine embryos during the fourth cell cycle after using PRP. That study (1992) corroborates with new findings (Farimani *et al*., 2016) that demonstrate the PRP method reduces abortion rates by increasing endometrial thickness.

In 2014, the first clinical application of PRP for mastitis using intramammary administration was reported, and the authors concluded that PRP might be useful for a quick resolution of the inflammatory response by playing a role in limiting the tissue damage of the mammary gland parenchyma and reducing the recurrence rates (Lange-Consiglio *et al*., 2014). In 2015, Lange-Consiglio and colleagues focused on reproductive problems by evaluating the effects of intrauterine administration of PRP on in vitro embryo production, with encouraging results. 

Marini *et al*. (2016) evaluated the effect of PRP *in vitro* for bovine endometrial inflammation and obtained an important anti-inflammatory response in the evaluated cells, concluding that PRP should be considered a potential treatment for endometritis in vivo. Endometritis is characterized by an increased number of inflammatory cells associated with epithelial erosion and/or necrosis, and diffuse oedema of the endometrium. In most cases, the normal uterus is able to clear a bacterial infection efficiently. However, an uncontrolled infection (10%-20% of cows) may lead to chronic uterine inflammation. 

Meta-analysis studies showed that endometritis reduces the pregnancy rate by 16% (Fourichon *et al*., 2000), and the economic losses related to this disease are substantial (Sheldon *et al*., 2009).

Jang *et al*. (2017) investigated PRP treatment for damaged endometrium, and the research group concluded that the intrauterine administration of autologous PRP stimulated and accelerated regeneration of the endometrium and decreased fibrosis in a murine model. 

With these animal model studies, we can conclude that PRP enriches the uterine environment with the growth factors necessary for embryo development and counteracts eventual subclinical endometritis by its anti-inflammatory properties.

## PRP and human reproduction

Since the first in vitro fertilization (IVF) attempts in the mid-1970s, researchers have been aware of the important role played by the endometrium, in addition to the embryo itself, in achieving a pregnancy (Bos-Mikich *et al*., 2018). Czernobilsky (1978) published a paper emphasizing the importance of the diagnosis of endometritis and its consequences on a woman’s fertility. 

Koot *et al*. (2011) attributed a successful implantation to a receptive endometrium, a functional embryo at the blastocyst developmental stage, and a synchronized dialogue between the maternal and embryonic tissues, in a perfect endometrial-embryo relationship. Recurrent implantation failures can be attributed to many factors, with one of the most important being a refractory endometrium, which is characterized by measurements below 6 mm and the presence of intrauterine adhesions (detected by a hysteroscopy). The causes can be idiopathic, congenital, or surgical (curettage), or by many inflammatory processes, infections, radiations, and other reasons (Coughlan *et al*., 2014; Kasius *et al*., 2014). 

The standard protocol for preparing infertile women’s endometria are hormone therapies. However, because of the complexity of causes, other therapies may be considered to ensure adequate synchronization. Recently, alternative treatment modalities have been explored, including growth factors (mainly G-CSF), stem cells, PRP, and bone marrow (Ohl *et al*., 2002; Barad *et al*., 2014; Kunicki *et al*., 2014; Singh *et al*., 2014; Chang *et al*., 2015).

A proportion of women has ‘unexplained’ infertility, in which pregnancies fail before they are clinically recognized. Garcia-Velasco *et al*. (2016) performed an extensive literature review on the management of refractory endometrium using conventional hormone protocols and autologous preparations as growth factors, such as PRP, and showed that the uterine environment is a protagonist in the process of embryonic failure. 

In 2015, Chang *et al*. performed a first clinical trial using PRP to improve endometrial thickness in patients undergoing IVF treatment. Five patients, with poor endometrial response after standard hormone replacement therapy, received an intrauterine infusion of PRP, 1-2 times in each cycle. The endometrial thickness increased, and four patients had a successful pregnancy, which proved PRP’s ability to improve pregnancy outcomes. Since then, some clinical studies and several reviews have explored the subject and concluded that intrauterine PRP therapy is promising, and the results are extremely encouraging.

Farimani *et al*. (2016) performed a single-blind pilot study to support the hypothesis that intrauterine administration of PRP could improve pregnancy outcomes of frozen-thawed embryo transfer. Nine patients with a history of recurrent implantation failure were selected for the study, and six women achieved clinical pregnancy (pregnancy rate was 66.6%). In 2017, Farimani reported a successful case of a 45 year-old woman with primary infertility and two failed IVF cycles. The patient was treated with intrauterine administration of autologous PRP to improve endometrial receptivity, and the patient became pregnant and had a term gestation, showing the effectiveness of PRP.

Zadehmodarres *et al*. (2017) in a pilot study recruited ten patients with a history of inadequate endometrial growth in frozen-thawed embryo transfer cycles. Intrauterine PRP infusion was successful for endometrial growth (adequate endometrial growth was found in all the participants after two PRP infusions).

Molina *et al*. (2018) also performed a clinical trial using PRP to increase endometrial quality and implantation rates in patients with refractory endometrium. Nineteen patients received an intrauterine infusion, and 73.7% became pregnant. The authors concluded that, despite the vast array of resources available today, it is still not easy to provide a pragmatic evidence-based approach that guides the clinician on how to improve refractory endometrium.

## Conclusions

PRP represents a novel strategy for reproductive medicine with clinical issues, such as thin endometrium (with poor response to conven tional therapy), as well as to obtain positive clinical pregnancies and live births. Being an autologous resource, PRP is harmless to the patient, easy to obtain, and of very low cost. Therefore, this protocol should be included for endometrial preparation in assisted reproduction techniques. However, more randomized, controlled trials with large sample sizes are necessary in this field. These studies and basic research on the cellular and molecular levels can improve our knowledge on PRP’s mode of action to better understand how and in what clinical situations it should be administered.
